# Fast dehydration reduces bundle sheath conductance in C_4_ maize and sorghum

**DOI:** 10.1111/nph.20167

**Published:** 2024-10-25

**Authors:** Chandra Bellasio, Hilary Stuart‐Williams, Graham D. Farquhar, Jaume Flexas

**Affiliations:** ^1^ Laboratory of Theoretical and Applied Crop Ecophysiology, School of Biology and Environmental Science University College Dublin Belfield Dublin 4 D04V1W8 Ireland; ^2^ Department of Chemistry, Biology and Biotechnology Università Degli Studi Di Perugia Perugia 06123 Italy; ^3^ Biology of Plants Under Mediterranean Conditions, Department of Biology University of the Balearic Islands 07122 Palma Illes Balears Spain; ^4^ Research School of Biology Australian National University Acton ACT 2601 Australia; ^5^ Agro‐Environmental and Water Economics Research Institute (INAGEA), Complex Balear de Recerca, Desenvolupament Tecnològic i Innovació (Parc Bit) Carrer Blaise Pascal, 6 07120 Palma Illes Balears Spain

**Keywords:** carbon reaction, drought, isotopic discrimination, limitation, model, photosynthesis

## Abstract

- In the face of anthropogenic warming, drought poses an escalating threat to food production. C_4_ plants offer promise in addressing this threat. C_4_ leaves operate a biochemical CO_2_ concentrating mechanism that exchanges metabolites between two partially isolated compartments (mesophyll and bundle sheath), which confers high‐productivity potential in hot climates boosting water use efficiency. However, when C_4_ leaves experience dehydration, photosynthesis plummets. This paper explores the physiological mechanisms behind this decline.
- In a fast dehydration experiment, we measured the fluxes and isotopic composition of water and CO_2_ in the gas exchanged by leaves, and we interpreted results using a novel biochemical model and analysis of elasticity.
- Our findings show that, while CO_2_ supply to the mesophyll and to the bundle sheath persisted during dehydration, there was a decrease in CO_2_ conductance at the bundle sheath‐mesophyll interface.
- We interpret this as causing a slowdown of intercellular metabolite exchange – an essential feature of C_4_ photosynthesis. This would impede the supply of reducing power to the bundle sheath, leading to phosphoglycerate accumulation and feedback inhibition of Rubisco carboxylation. The interplay between this rapid sensitivity and the effectiveness of coping strategies that C_4_ plants deploy may be an overlooked driver of their competitive performance.

## Introduction

**Fig. 1:**
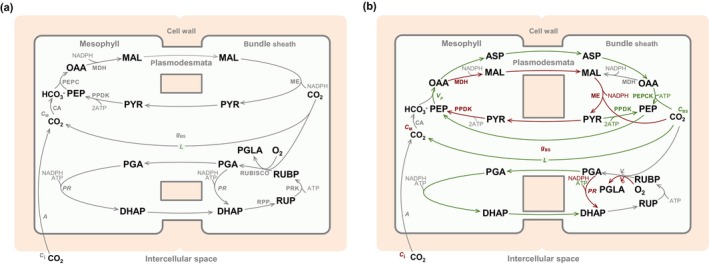
(a) Schematic of two‐celled C_4_ photosynthesis. The leaf parenchyma differentiates two types of cells, the mesophyll and the bundle sheath, exchanging metabolites through plasmodesmata to operate a CO_2_ concentrating mechanism. CO_2_ entering the mesophyll is hydrated to bicarbonate by carbonic anhydrase (CA) and fixed by phosphoenolpyruvate (PEP) carboxylase (PEPC) to form oxaloacetate (OAA). OAA may be reduced by malate dehydrogenase (MDH) to malate (MAL) which diffuses to the BS through plasmodesmata. MAL is decarboxylated mainly by malic enzyme (ME) to pyruvate (PYR), delivering NADPH and concentrating CO_2_ in the BS. PYR diffusing back to the M is reconverted into PEP by pyruvate‐phosphate‐dikinase (PPDK). Rubisco carboxylation and oxygenation reactions, fully compartmentalised in bundle sheath cells, consume ribulose 1,5‐bisphosphate (RuBP) and produce phosphoglycerate (PGA) and phosphoglycolate (PGLA). PGA is reduced at a rate *PR* to triose phosphate (DHAP), which is a substrate of carbohydrate synthesis (not shown), the final product of photosynthesis. The majority of DHAP enters the sugar conversion phase of the reductive pentose phosphate (RPP) cycle regenerating ribulose 5‐phosphate (RuP), the substrate of phosphoribulokinase (PRK). The photorespiration cycle regenerating PGLA to PGA is not shown. (b) Interpretation of responses to dehydration. Red indicates quantities predicted to have sharper decrease (more negative elasticity) than CO_2_ assimilation (*A*) during dehydration. Green represents lesser decrease (higher elasticity) than assimilation, while grey arrows denote elasticity similar to assimilation. Reduced bundle sheath conductance (*g*_BS_) can hinder MAL diffusion to the BS slowing down NADPH production by ME. This, in turn, constrains the BS's capacity to reduce PGA (*PR*); PGA may accumulate, inhibiting Rubisco carboxylation (*V*_C_). The excess PGA in the BS would diffuse to the mesophyll, consuming ATP and limiting PPDK activity in the mesophyll. The surplus ATP in the bundle sheath would be used to regenerate PEP, either by PEP carboxykinase (PEPCK) or by PPDK (using PYR derived from a futile oxydoreduction of OAA catalysed by MDH), sustaining the rate of PEP carboxylase (*V*_P_). The additional OAA produced in the mesophyll would be transaminated to an alternative shuttle through aspartate (ASP), which would deliver CO_2_ to the BS without requiring NADPH in the M. This would increase CO_2_ concentration in the BS (*C*_BS_) and suppress Rubisco oxygenation (*V*_O_). *C*_i_ and *C*_M_ are the CO_2_ concentrations in the intercellular spaces and in the mesophyll, respectively. Amino‐groups are rebalanced between mesophyll and bundle sheath through transamination of pyruvate (not shown).

Drought has a devastating impact on crops, leading to food shortages, and economic hardships, which are expected to be exacerbated in the future (Naumann *et al*., 2021). Due to their high productivity in warm climates, heat resilience, and efficient water use, C_4_ plants are promising options to boost crop production, amidst population pressure and anthropogenic warming (Furbank *et al*., 2015). While the ancestral C_3_ photosynthesis happens within each chloroplast, C_4_ photosynthesis is shared between mesophyll and bundle sheath cells (Fig. 1a). These are coupled to operate a biochemical CO_2_ concentrating mechanism that effectively pumps CO_2_ to the sites of carboxylation (Hatch, 1987). CO_2_ is hydrated to bicarbonate and fixed by the fast and specific enzyme phosphoenolpyruvate carboxylase (PEPC) in the mesophyll (Bellasio & Farquhar, 2019). The resulting four‐carbon (C_4_) organic acids diffuse to the bundle sheath through plasmodesmata (their permeability to CO_2_ at leaf level is measured by bundle sheath conductance, *g*_BS_). The decarboxylation of C_4_ acids delivers and concentrates CO_2_ around Rubisco, favouring carboxylation over oxygenation and thereby suppressing energy costly photorespiration (von Caemmerer & Furbank, 2003; Bellasio *et al*., 2014). About half of the phosphoglycerate (PGA) produced by Rubisco and all the pyruvate (PYR) resulting from C_4_ acid decarboxylation diffuses back to the mesophyll (Bellasio, 2017). Phosphoglycerate is then chemically reduced and diffuses back to the bundle sheath to complete the reductive pentose phosphate (RPP) cycle. This prolific exchange of metabolites is essential for C_4_ photosynthesis to work (Weber & von Caemmerer, 2010).

Classically, in nondormant plants, resistance to drought has been categorised into avoidance and tolerance strategies, that is, minimising exposure *vs* capacity to withstand (for review see Bacelar *et al*., 2012). The C_4_ cycle drives steep CO_2_ concentration gradients across stomata, allowing high rates of assimilation with low transpiration. This prolongs soil moisture retention, delaying stress when C_4_ plants become water limited, making C_4_ plants superb drought avoiders. In typical irrigation withdrawal experiments, avoidance can often be confused with tolerance if the time since reduced watering is taken as a proxy for stress intensity. To distinguish between these two strategies, stress intensity should be measured in terms of leaf water potential (Ψ_L_), and the reciprocal of tolerance can be quantified as the relative response divided by the absolute or relative change in Ψ_L_, referred to as sensitivity or elasticity, respectively (Bellasio *et al*., 2023). Sensitivity to dehydration is generally more pronounced for C_4_ assimilation than C_3_, especially when dehydration is rapid. Takeda *et al*. (1983) dehydrated leaf discs of 76 species using sorbitol, showing in 14 C_4_ grasses and 16 C_4_ *Cyperaceae* species that 50% reduction in assimilation occurred at a much less negative Ψ_L_ of −1.5 MPa compared to −3.8 MPa in 19 C_3_ grass species and 27 C_3_ *Cyperaceae* species. On intact plants grown in hydroponics we observed that assimilation started to decline at a Ψ_L_ that was less negative in C_4_ maize and sorghum than in C_3_ wheat and sunflower (Bellasio *et al*., 2023). In a controlled environment, we showed in three grasses that C_4_ assimilation declined between subsequent watering intervals with corresponding modest dips in Ψ_L_ that did not trigger stress indicators or affect assimilation in C_3_ plants (Quirk *et al*., 2019). In comparative field studies, Taylor *et al*. (2014) found that C_4_ species, but not C_3_, experienced water limitation in correspondence to midday depressions of Ψ_L_. In natural field conditions across a transect in Western China, encompassing a variety of climates and elevations, we found that five C_4_ Amaranthaceae species well‐adapted to their environment had a greater depression of photosynthesis due to water limitation compared to their C_3_ neighbours (Flexas *et al*., 2022).

Stomatal closure, restraining the supply of CO_2_ to the leaf, is not the key impediment to C_4_ photosynthesis during dehydration (Ghannoum *et al*., 2003; Carmo‐Silva *et al*., 2007, 2008; Ripley *et al*., 2007, 2010). Evidence for the dominance of other nonstomatal factors was gained both through gas exchange analyses, as in our studies (Bellasio *et al*., 2018), and by bypassing stomatal limitation, as in the *in vitro* experiment of Takeda *et al*. (1983) where bicarbonate concentration was presumably saturating (Ishii *et al*., 1977; Takeda, 1984). Under rapid dehydration nonstomatal limitation in C_4_ maize and sorghum rose three times more steeply than in C_3_ wheat and sunflower (Bellasio *et al*., 2023). In three grasses from each of the different C_4_ subtypes, the carboxylation rate of Rubisco declined much more rapidly than that of PEP carboxylase (Carmo‐Silva *et al*., 2008). Rather than permanent enzyme deactivation, these processes appeared to be regulated by a rapid mechanism, potentially impairing the balance between the C_4_ and the C_3_ cycle (Ghannoum, 2009). However, the underpinning physiological mechanisms remained unknown.

The aim of this study was to identify the cause of the sensitivity of C_4_ assimilation to dehydration. We hypothesise that the C_4_ machinery could jam due to structural alterations that may hinder the delivery of CO_2_ to the mesophyll or the intercellular exchange of metabolites between the mesophyll and bundle sheath. The roots of hydroponically grown plants were drawn out of water to induce a fast but controllable dehydration over a timescale of hours (Supporting Information Fig. S1). Gas exchange, Chl fluorescence, as well as the isotopic composition of CO_2_ and water were monitored concurrently in real time. A biochemical model of C_4_ electron transport and carbon reactions, purposely formulated for this study, was coupled to a model of isotopic discrimination to resolve mesophyll and bundle sheath conductances to CO_2_. We test whether nonstomatal limitation is associated with a decrease in mesophyll conductance (Hypothesis 1) or bundle sheath conductance (Hypothesis 2).

## Materials and Methods

### Plants

Seeds of *Zea mays* L, and *Sorghum bicolor* (L) Munch, were germinated for a week on wet paper. Twenty‐litre black polypropylene tubs filled with water were fertilised with 150 cm^3^ of Green Dream 1 complete fertiliser (Flairform, Applecross, WA, Australia), supplemented with 2 g of Fe‐EDTA for maize. Seedlings were transferred in foam rubber discs placed in 5 cm holes cut in the lids of the tubs. The solution was constantly aerated through aquarium stones, fertilised weekly with 50 cm^3^ of the mentioned fertiliser and discarded after 3 wk. Plants were grown for 4–6 wk in controlled environment plant growth chambers (Thermoline Scientific, Wetherill Park, NSW, Australia), set at 26°C : 20°C (day : night), 80% relative humidity, with a 12‐h photoperiod inclusive of a 9‐h day (400 μmol m^−2^ s^−1^) interrupted by a 1‐h midday peak illumination (690 μmol m^−2^ s^−1^ 1000 W metal halide arc lamps multi vapor® MVR; GE Lighting, East Cleveland, OH, USA, plus halogen), and flanked by 1‐h dawn and 1‐h dusk (80 μmol m^−2^ s^−1^ only halogen).

### Instrumental setup

Leaf exchange of CO_2_ and H_2_O were measured with a portable system (LI6400XT; Li‐Cor, Lincoln, NE, USA), modified to operate at low‐CO_2_ concentrations, and fitted with a 6400‐06 PAM2000 adapter, holding a glass fibre probe in the upper leaf cuvette, oriented to minimise shading of the leaf (Fig. S1). The fibre was connected to a Dual PAM–F (Heinz Walz GmbH, Effeltrich, Germany) to measure photosystem II yield. Pulse intensity was adjusted to be between 10 000 and 12 000 μmol m^−2^ s^−1^ thereby exceeding the saturating intensity of *c*. 8000 μmol m^−2^ s^−1^. Neoprene gaskets were used on both sides of the cuvette; CO_2_ diffusion through the gaskets was compensated by lengthening the tubing of the LI6400XT reference gas, so that the total amount of CO_2_ diffusing into the reference and sample sides was similar. Light was provided by a 6400‐18 RGB light source positioned to illuminate the leaf uniformly. Light intensity was measured by the gallium arsenide photodiode in the light source, repositioned in the leaf chamber, and calibrated using a Li‐250 light sensor (Li‐Cor).

Synthetic air was prepared by mixing O_2_ and N_2_ with a bespoke gas mixing system consisting of rapid response mass flow controllers (Alicat Scientific, Tucson, AZ, USA), controlled by a Raspberry Pi 3 (raspberrypi.org) and a custom interface program by HS‐W (vinland) operating on Microsoft Windows®. The synthetic air was humidified to a dew point of *c*. 17°C upstream of the LI6400XT inlet to maintain in the cuvette a water vapour pressure deficit of *c*. 1 kPa. Laser Quality CO_2_, with δ^13^C = −7.02‰ V‐PDB by measurement against a multiply calibrated CO_2_ (TUCK) using a dual‐inlet Isotope Ratio Mass Spectrometer (IRMS) and a modified Continuous Flow‐IRMS, was added from a cylinder (BOC, North Ryde, Australia), using the CO_2_ injection unit of the LI6400XT.

The gas flow was split into three lines. The first was connected to the LI6400XT reference and sample cuvette. The second to a cavity‐ring down absorption spectrometer (L‐1102i; Picarro Inc., Sunnyvale, CA, USA), measuring the ^1^H_2_O^16^ and ^1^H_2_O^18^ isotopologues of H_2_O. The L‐1102 was periodically calibrated; this was done with an in‐house capillary nebuliser consisting of a pressurised water cylinder feeding reference water, calibrated against International Atomic Energy Agency standard waters V‐SMOW and VSLAP, onto the centre of an electric computer fan in a sealed canister with dry nitrogen flow. Reference water is instantly vapourised so that moisture in the air has a composition identical to that of the standard. The third stream of reference gas was Nafion‐dried and connected to a Quantum‐Cascade Laser system (QCL; Aerodyne Research, Billerica, MA, USA), measuring specifically the CO_2_ isotopologue composition (concentration of ^12^C^16^O_2_, ^13^C^16^O_2_ and ^12^C^18^O^16^O).

The sample gas was the LI6400XT cuvette exhaust diverted from the matching tube and split into two lines, which were fed to the L‐1102i or Nafion‐dried and fed to the QCL. The CO_2_ concentration of the reference gas was matched to that of the sample by diluting with synthetic air taken from upstream of the LI6400XT CO_2_ mixing unit, with the rate controlled using a micrometric needle valve. Data from the QCL were collected by vinland through an Arduino Mega (arduino.org). To mitigate laser influences, including foreign broadening, Vinland automated sample analysis correction through standardised routines alternating between sample and reference gas. The laser response is linear over this range and gives excellent precision, which was typically < 0.2‰ for δ^18^O.

### Gas exchange, fluorescence, and isotopic discrimination during dehydration

The evening before the experiment, plants were bagged in the dark and transferred to the laboratory. A fully expanded leaf was fitted with the PSY1 thermocouple of a PSY1 psychrometer (ICT, Armidale, NSW, Australia), calibrated with five standard NaCl solutions. A small window of epidermis was removed with a razor blade from a fully expanded leaf of a plant standing in aerated water, rinsed with abundant distilled water. This was blotted with paper and fitted with the thermocouple of the PSY1, then sealed with a tiny ridge of high‐vacuum grease following the manufacturer's instructions. An adjacent portion of the leaf was clamped in the cuvette of the LI6400XT. Plants were acclimated in the dark overnight with ambient air supply. In the morning, *PPFD* was set at 500 μmol m^−2^ s^−1^, reproducing growth *PPFD*, air supply was switched to synthetic air either with ambient or 2% [O_2_] with a cuvette flow rate of 490 μmol s^−1^, and reference [CO_2_] of 300 μmol mol^−1^, which maximised *C*_i_ : *C*_a_. After photosynthetic induction for a minimum of 1 h, every 15–20 min, assimilation, Ψ_L_, PSII yield were measured both under ambient and 2% [O_2_], while isotopic composition was measured under ambient [O_2_]. Roots were progressively exposed by lowering the water bucket (Fig. S1), with the aim of reducing Ψ_L_ in steps of 0.1–0.15 MPa for each measurement period, until leaves were irreversibly wilted, after 6–8 h.

vinland calculates CO_2_ isotopic composition δ^13^C and δ^18^O (eqn 4 in Ubierna *et al*., 2018b), following the method of Griffith *et al*. (2012). The resulting CO_2_ δ^18^O V‐PDB is converted to V‐SMOW as: δ^18^O(V‐SMOW) = 1.03092 × δ^18^O(V‐PDB) + 30.92 (Brand *et al*., 2014). The L‐1102 calculates isotopic composition (δ^2^H and δ^18^O V‐SMOW) directly from its laser measurements and returns these to the user. Isotope discrimination (the change in isotopic composition in ambient air induced by the plant during photosynthesis, eqn 3 in Ubierna *et al*., 2018b) measured for ^13^C ($\Delta_{\mathrm{OBS}}^{13}$) or ^18^O ($\Delta_{\mathrm{OBS}}^{18}$) was calculated as per Evans *et al*. (1986):

(Eqn 1) $$\Delta_{\mathrm{OBS}}=\frac{1000\;\frac{C_{\text{in}}}{C_{\text{in}}-C_{\text{out}}}\;\left(\delta_{\text{out}}-\delta_{\text{in}}\right)}{1000+\delta_{\text{out}}-\frac{C_{\text{in}}}{C_{\text{in}}-C_{\text{out}}}\;\left(\delta_{\text{out}}-\delta_{\text{in}}\right)}$$

and isotopic discrimination of ^18^O ($\Delta^{18}O$) was calculated analogously, where *C* is the ^12^CO_2_ mole fraction in dry air in and out of the chamber, and *δ* refers to either the δ^13^C or δ^18^O.

### Rate of ATP production

The total ATP production rate was calculated for each measured datapoint, using *R*_LIGHT_ (respiration in the light) and scaling assimilation measured under low [O_2_] to the increase in *Y*(II) measured between low and ambient [O_2_] after Bellasio & Griffiths (2014a) as:

(Eqn 2) $$J_{\mathrm{ATP}}=5.4\;{\mathrm{GA}}_{\mathrm{Low}\;O_{2}}\;\frac{\;Y\left(\mathrm{II}\right)\;}{Y{\left(\mathrm{II}\right)}_{\mathrm{Low}\;O_{2}}}$$

where 5.4 is the ATP cost of gross assimilation under low [O_2_] assumed as the lowest value that gave positive solutions for CO_2_ concentration in the bundle sheath, *C*_BS_ (Eqn 6), GA_LOW_ is gross assimilation measured under low O_2_ (GA = *A* + *R*_LIGHT_), *R*_LIGHT_ (1.19 for maize and 0.92 μmol m^−2^ s^−1^) was previously determined on *n* = 4 biological replicates as per Bellasio *et al*. (2016); *Y*(II) and $Y{\left(\mathrm{II}\right)}_{\mathrm{Low}\;O_{2}}$ is PSII yield under ambient and low [O_2_], respectively.

### CO_2_ concentration at the mesophyll carboxylation sites C_M_

Δ^18^O and Δ^13^C were combined with the concentration of ^1^H_2_O^16^ and ^1^H_2_O^18^ isotopologues of H_2_O to calculate the CO_2_ concentration at the mesophyll carboxylation sites (*C*_M_) after Ubierna *et al*. (2017) as:

(Eqn 3) $$C_{M}=C_{i}\frac{\;\delta_{i}^{18}-\alpha_{w}^{18}{\;\delta }_{A}^{18}-{\;\alpha }_{w}^{18}\;}{\delta_{\mathrm{ce}}^{18}-\alpha_{w}^{18}{\;\delta }_{A}^{18}-\alpha_{w}^{18}}$$

where *C*_i_ is the CO_2_ concentration in the intercellular spaces recalculated from data outputted by the LI6400XT using the method of Márquez *et al*. (2021), setting cuticular conductance to water vapour at 2 mmol m^−2^ s^−1^ (Boyer & Kawamitsu, 2011) and the ratio between cuticula conductance to water and CO_2_ (β) at 0.025 (Márquez *et al*., 2021); $\delta_{i}^{18}$ is the δ^18^O of the CO_2_ in the intercellular spaces (Cernusak *et al*., 2004; Farquhar & Cernusak, 2012) calculated as given in table 3 in Ubierna *et al*. (2017); $\alpha_{w}^{18}=1+\frac{a_{w}^{18}}{1000}$, where $a_{w}^{18}$ is the summed discrimination against C^18^O^16^O during liquid phase diffusion and dissolution (0.8‰), ${\;\delta }_{A}^{18}$ is the δ^18^O of the CO_2_ taken up by photosynthesis (Evans *et al*., 1986; Cernusak *et al*., 2004) calculated as given in table 3 in Ubierna *et al*. (2017); $\delta_{\mathrm{ce}}^{18}$ is the δ^18^O of the CO_2_ in equilibrium with cytosol water (Cernusak *et al*., 2004) calculated by eqn 11 in Ubierna *et al*. (2017); parameterisation was taken from Ubierna *et al*. (2017). Mesophyll conductance to CO_2_ diffusion (*g*_M_) was calculated from the mesophyll supply function: $g_{M}=\frac{A}{C_{i}-C_{M}}$.

### Reaction rates and bundle sheath conductance

We derived a new formulation of a C_4_ model (Table 1). Net assimilation is (Farquhar *et al*., 1980):

(Eqn 4) $$A=V_{C}-0.5\;V_{O}-R_{\text{LIGHT}}$$

where *V*_C_ and *V*_O_ are Rubisco rate of carboxylation and oxygenation. *V*_O_ is expressed as a function of the CO_2_ and the O_2_ concentration in the bundle sheath, *C*_BS_ and *O*_BS_, respectively (von Caemmerer, 2000) as:

(Eqn 5) $$V_{O}=2\;\gamma^{*}\;\frac{O_{\mathrm{BS}}}{C_{\mathrm{BS}}}$$

where γ* is half the reciprocal Rubisco specificity, 0.000233 (Cousins *et al*., 2010).

**Table 1:** Comparison between model fitting strategies to derive *g*_BS_.

	*J*/*J* Fitting	Δ/Δ Fitting	New approach
Degrees of freedom	(16): *A*, *R*_LIGHT_, *J*_ATP_, *V*_C_, *V*_P_, *V*_O_, *C*_BS_, *O*_BS_, $\gamma^{*}$, *C*_M_, *R*_M_, α, *O*_M_, *x*, *g*_BS_, *g*_M_	(25): *A*, *R*_LIGHT_, *J*_ATP_, *V*_C_, *V*_P_, *V*_O_, *C*_BS_, *O*_BS_, $\gamma^{*}$, *C*_M_, *R*_M_, α, *O*_M_, *x*, *g*_BS_, Δ_MOD_, *t*, *C*_a_, *C*_i_, *a*_S_ *a*_m_, *b*_4_, *b*_3_, *s*	(23): *A*, *R*_LIGHT_, *J*_ATP_, *V*_C_, *V*_P_, *V*_O_, *C*_BS_, *O*_BS_, $\gamma^{*}$, *C*_M_, *R*_M_, α, *O*_M_, *g*_BS_, Δ_MOD_, *t*, *C*_a_, *C*_i_, *a*_S_, *a*_m_, *b*_4_, *b*_3_, *s*
*Constraints*			
Equations	(6): $A=V_{C}-0.5\;V_{O}-R_{\text{LIGHT}}$; $V_{O}=2\;\gamma^{*}\;\frac{O_{\mathrm{BS}}}{C_{\mathrm{BS}}}$; $C_{\mathrm{BS}}=C_{M}+\frac{\;V_{P}-R_{M}-A\;}{g_{\mathrm{BS}}}$; $O_{\mathrm{BS}}=O_{M}+\frac{\alpha \;A}{0.047\;g_{\mathrm{BS}}}$; $V_{P}=\frac{x\;J_{\mathrm{ATP}}}{2}$; $C_{M}=C_{i}-\frac{A}{g_{M}}$.	(7): $A=V_{C}-0.5\;V_{O}-R_{\text{LIGHT}}$; $V_{O}=2\;\gamma^{*}\;\frac{O_{\mathrm{BS}}}{C_{\mathrm{BS}}}$; $C_{\mathrm{BS}}=C_{M}+\frac{\;V_{P}-R_{M}-A\;}{g_{\mathrm{BS}}}$; $O_{\mathrm{BS}}=O_{M}+\frac{\alpha \;A}{0.047\;g_{\mathrm{BS}}}$; $V_{P}=\frac{x\;J_{\mathrm{ATP}}}{2}$; $C_{M}=C_{i}-\frac{A}{g_{M}}$; $\Delta_{\mathrm{MOD}}=f\left(t,C_{a},C_{i},C_{M},C_{\mathrm{BS}},V_{P},R_{M},A,a_{S},a_{m},b_{4},b_{3},s\right)$	(6): $A=V_{C}-0.5\;V_{O}-R_{\text{LIGHT}}$; $V_{O}=2\;\gamma^{*}\;\frac{O_{\mathrm{BS}}}{C_{\mathrm{BS}}}$; $C_{\mathrm{BS}}=C_{M}+\frac{\;V_{P}-R_{M}-A\;}{g_{\mathrm{BS}}}$; $O_{\mathrm{BS}}=O_{M}+\frac{\alpha \;A}{0.047\;g_{\mathrm{BS}}}$; $J_{\mathrm{ATP}}=3\;V_{C}+3.5\;V_{O}+2\;V_{P}$; $\Delta_{\mathrm{MOD}}=f\left(t,C_{a},C_{i},C_{M},C_{\mathrm{BS}},V_{P},R_{M},A,a_{S},a_{m},b_{4},b_{3},s\right)$
Measured data	(3): *A*, *R*_LIGHT_, *C*_i_	(6): *A*, *R*_LIGHT_, *J*_ATP_, *C*_a_, *C*_i_, *t*	(7): *A*, *R*_LIGHT_, *J*_ATP_, *C*_a_, *C*_i_, *C*_M_, *t*
From the literature	(2): $\gamma^{*}$; $g_{M}$	(7): $\gamma^{*}$, *a*_S_, *a*_m_, *b*_4_, *b*_3_, *s*; $g_{M}$	(6): $\gamma^{*}$, *a*_S_, *a*_m_, *b*_4_, *b*_3_, *s*
Assumptions	(4): *x* = 0.4, *R*_M_ = 0.5*R*_LIGHT_, *O*_M_ = ambient, α = 0.15	(4): *x* = 0.4, *R*_M_ = 0.5*R*_LIGHT_, *O*_M_ = ambient, α = 0.15	(3): *R*_M_ = 0.5*R*_LIGHT_, *O*_M_ = ambient, α = 0.15
Dummy variable	(1): *g*_BS_	(1): *g*_BS_	(1): *g*_BS_
Fitting strategy	Modelled *J*_ATP_ to measured *J*_ATP_	Δ_MOD_ (Eqn 9) to measured Δ_OBS_ (Eqn 1)	Δ_MOD_ (Eqn 9) to measured Δ_OBS_ (Eqn 1)

Previously, we proposed two alternative fitting approaches that involved either fitting the model to *J*_ATP_ (*J*/*J* fitting) or to Δ^13^C isotopic discrimination data (Δ/Δ fitting) (Bellasio & Griffiths, 2014a). That innovation constrained the C_4_ model better than is achieved with the method of Ubierna *et al*. (2011), because *J*_ATP_ was derived based on fluorescence data and measurements of assimilation under both ambient and low [O_2_], but had the shortcoming of outputting two separate values for *g*_BS_. Here, we combined five validated equations to derive a single C_4_ model constrained with measurements of *C*_M_ and *J*_ATP_. Then, we modelled isotopic discrimination after Farquhar & Cernusak (2012) (simplified in the table as *f*(…)) and estimated a unified *g*_BS_ by iteratively fitting modelled Δ^13^C to measured Δ^13^C data. Numbers in brackets count degrees of freedom and constraints.

The CO_2_ supply function for the bundle sheath is (Berry & Farquhar, 1978):

(Eqn 6) $$C_{\mathrm{BS}}=C_{M}+\frac{\;V_{P}-R_{M}-A\;}{g_{\mathrm{BS}}}$$

where *V*_P_ is PEPC rate of carboxylation, *R*_M_ is respiration in the mesophyll taken as half of *R*_LIGHT_ (von Caemmerer, 2000), *g*_BS_ is bundle sheath conductance to CO_2_, and the O_2_ concentration in the bundle sheath is (Berry & Farquhar, 1978):

(Eqn 7) $$O_{\mathrm{BS}}=O_{M}+\frac{\alpha \;A}{0.047\;g_{\mathrm{BS}}}$$

where *O*_M_ is the O_2_ concentration in the mesophyll, assumed to equal ambient, 0.047 scales O_2_ to CO_2_ diffusivity and solubility (Berry & Farquhar, 1978) and α, here 0.15 (Bellasio & Griffiths, 2014a), scales the O_2_ evolution in the bundle sheath.

At steady state, when respiration is supplied from new assimilates and the ATP cost of carbohydrate synthesis is neglected, *J*_ATP_ is (Bellasio, 2017):

(Eqn 8) $$J_{\mathrm{ATP}}=3\;V_{C}+3.5\;V_{O}+2\;V_{P}$$

Eqns (Eqn 1), (Eqn 2), (Eqn 3), (Eqn 4), (Eqn 5), (Eqn 6), (Eqn 7), (Eqn 8) form a system of five equations with five unknowns (*V*_C_, *V*_P_, *V*_O_, *C*_BS_, and *O*_BS_), which were solved in matlab for *V*_C_, *V*_P_ and *V*_O_ as reported in Notes S1. The three solutions, together with Eqns 6 and 7, output *V*_C_, *V*_P_, *V*_O_, *C*_BS_, and *O*_BS_ using measured *A*, *J*_ATP_ (Eqn 2), and *C*_M_ (Eqn 3).

Modelled isotopic discrimination was calculated after Farquhar & Cernusak (2012), in the formulation of Ubierna *et al*. (2018a) as:

(Eqn 9) $$\Delta_{\mathrm{MOD}}=\begin{array}{l} \frac{1}{1-t}a_{S}\frac{C_{a}–C_{i}}{C_{a}}+\frac{1+t}{1-t}[a_{m}\frac{C_{i}–C_{M}}{C_{a}}\\ +\;\frac{b_{4}+\Phi \left(b_{3}\frac{C_{\mathrm{BS}}}{C_{\mathrm{BS}}–C_{M}}–s\right)}{1+\Phi \frac{C_{M}}{C_{\mathrm{BS}}–C_{M}}}\frac{C_{M}}{C_{a}}] \end{array}$$

Due to vigorous ventilation in the gas exchange cuvette, we were able to disregard fractionation at the boundary layer, as in Bellasio & Griffiths (2014a), *t* is the ternary effect Farquhar & Cernusak (2012), $t=\frac{\left(1+a_{S}\right)\;E}{2000\;g_{\mathrm{SC}}}$, *E* is leaf transpiration, *g*_SC_ is stomatal conductance to CO_2_, *a*_m_ represents the fractionation during dissolution of CO_2_ and diffusion of aqueous CO_2_ calculated after Farquhar (1983) in the formulation of Kromdijk *et al*. (2010); *a*_s_ is the fractionation during CO_2_ diffusion in air; *s* is the fractionation during CO_2_ leakage; *b*_3_ is the fractionation of Rubisco CO_2_ fixation, corrected for respiration and photorespiration; *b*_4_ is the combined fractionation of CO_2_ ↔ HCO_3_^−^ conversion and PEPC fixation, corrected for mitochondrial respiration in the mesophyll all calculated after Farquhar (1983), in the formulation of Bellasio & Griffiths (2014a); *f* the fractionation during photorespiration (Farquhar, 1983), was 11.6‰ (Lanigan *et al*., 2008); in this study we used CO_2_ from a single calibrated cylinder, δ^13^C_measurements_ = −7.02‰; and δ^13^C_growth chamber_ = −8‰.

We imposed a linear dependency of *g*_BS_ on Ψ_L_ within each daily replicate as:

(Eqn 10) $$g_{\mathrm{BS}}=g_{\mathrm{BS}\;0}+k\;\Psi_{L}$$

where *g*_BS 0_ is bundle sheath conductance at full hydration, and *k* represents the dependence of *g*_BS_ on Ψ_L_. *k* and *g*_BS0_ were concurrently found for each plant by iteratively fitting Eqn 10 to experimental data (Eqn 1) using Excel® Solver evolutionary algorithm (Frontline Systems, Incline Village, NV, USA) with default settings. The maximum time or number of iterations was not constrained: each fitting process consisted of *c*. 23 000 subproblems, each comprising 6000 to 10 000 iterations. We repeated the fitting of *g*_BS 0_ and *k* using a more limited range of datapoints, excluding either the last or the last two datapoints.

### Error propagation analysis

We utilised a Monte Carlo technique following Verbeeck *et al*. (2006), to calculate the model output uncertainty. As per Verbeeck *et al*. (2006) we did not differentiate between the uncertainty and natural variability of the parameters, and we did not consider the uncertainty of the measured input variables such as respiration and light intensity (Table S1). For each parameter, we generated 200 random parameter combinations, input them into the deterministic model, and found the values of *k* and *g*_BS0_ iteratively, as described above.

### Statistical analysis

To evaluate the effect of Ψ_L_ on measured and modelled quantities, log_e_‐transformed variables were subjected to a repeated measurement linear mixed model (REML, genstat) with log_e_(Ψ_L_) as a fixed factor and day as random factor. The slope of the regressions (elasticity, η_Ψ_) are in Table 2; the intercept was not used. The hypothesis of *k* not greater than zero was tested with a single sample *t*‐test in genstat (Table 3) or in excel (Table S2). We tested the effect of excluding the last datapoints of each day‐replicate with a general ANOVA where method (normal, discard last, discard two), species (maize, sorghum), and quantity (*g*_BS 0_, *k*) were fixed factors (genstat).

**Table 2:** Elasticity (or relative sensitivity) to dehydration.

Symbol	Unit	Description	Elasticity to dehydration (η_Ψ_, dimensionless)				
			Maize	Sorghum	Average	SED	Rank
*A*	μmol m^−2^ s^−1^	Assimilation rate	−0.33	−0.19	−0.26	n/a	5
*C*_BS_	μmol mol^−1^	CO_2_ concentration in the bundle sheath	1.2	0.30	0.75	0.24	13
*C*_i_	μmol mol^−1^	CO_2_ concentration in the substomatal cavity	−0.44	−0.32	−0.38	0.24	4
*C*_M_	μmol mol^−1^	CO_2_ concentration in the mesophyll	−0.49	−0.37	−0.43	0.22	3
*g*_BS_	mol m^−2^ s^−1^	Bundle sheath conductance to CO_2_	−1.2	−0.26	−0.73	0.27	1
*J*_ATP_	μmol m^−2^ s^−1^	Total ATP production rate	−0.28	−0.14	−0.21	0.27	7
*V*_C_	μmol m^−2^ s^−1^	Rubisco rate of carboxylation	−0.31	−0.18	−0.25	0.24	6
*V*_O_ */V*_C_	Dimensionless	Rubisco rate of carboxylation over oxygenation	−0.87	−0.29	−0.58	0.24	2
*V*_P_	μmol m^−2^ s^−1^	PEPC rate of carboxylation	−0.23	−0.086	−0.16	0.24	9
*Y*(II)	Dimensionless	Yield of Photosystem II	−0.2	−0.17	−0.19	0.24	8
Δ^13^C	‰	Carbon isotopic discrimination	0.49	0.12	0.31	0.27	12
Δ^18^O	‰	Oxygen isotopic discrimination	−0.21	−0.1	−0.16	0.24	10
Φ	Dimensionless	Leakiness, that is the rate of CO_2_ retrodiffusing from the bundle sheath to the mesophyll relative to *V*_P_	0.21	0.2	0.21	0.27	11

Elasticity was calculated as relative change in the variable, divided by the relative negative change in water potential $\eta_{\Psi }=-\frac{\mathrm{\partial Var}/\mathrm{Var}}{\partial \Psi_{L}/\Psi_{L}}$. The order (from low to high elasticity) is calculated on the absolute |η_Ψ_| averaged for the two species. The heat map visualises a gradient from the most positive elasticity in green to the most negative in red. The SE of the difference (SED) refers to the paired comparison with assimilation. The probability of η_Ψ_ = 0, obtained in a linear mixed model (REML) was *P* < 0.001 for all quantities except Sorghum *Y*(II) (*P* = 0.007).

**Table 3:** Quantities derived from model fitting from combined gas exchange, fluorometry, oxygen‐18 and carbon‐13 isotopic discrimination, and associated statistics.

Description	Symbol	Unit	Maize	*P*(*t*)	Sorghum	*P*(*t*)
Bundle sheath conductance at full hydration	*g*_BS0_	mol m^−2^ s^−1^	0.00209 ± 0.00026 (6)		0.00200 ± 0.00014 (7)	
Fitted dependency of *g*_BS_ on Ψ_L_	*k*	mol m^−2^ s^−1^ MPa^−1^	0.00218 ± 0.00039 (6)	0.001	0.00154 ± 0.00022 (7)	0.00005

Values are the average *g*_BS0_ and *k* ± SE obtained experimentally on replicated individual plants with initial parameterisation (Supporting Information Table S1); *P*(*t*) is the probability of *k* not greater than zero, tested for the number of biological replicates shown in brackets.

## Results

**Fig. 2:**
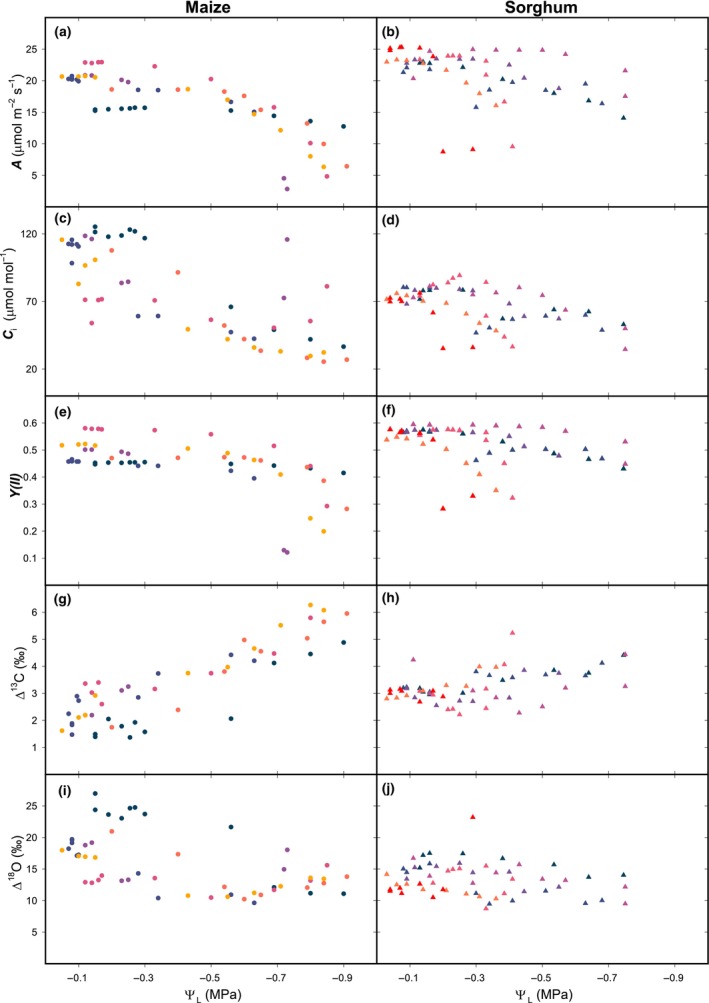
Concurrent gas exchange, fluorescence and isotopic discrimination. Maize (left) and sorghum (right) plants grown in hydroponics were progressively drawn out of the water while water potential (Ψ_L_) was measured at regular intervals, coupled with gas exchange data, fluorescence, carbon and oxygen isotopic discrimination (Δ^13^C and Δ^18^O, respectively), under ambient O_2_. (a, b) Assimilation (*A*); (c, d) CO_2_ concentration in the substomatal cavity (*C*_i_); (e, f) yield of PSII (*Y*(II)); (g, h), Δ^13^C and (i, j) Δ^18^O of CO_2_. Maize *n* = 6; sorghum *n* = 7 biological replicates, respectively; all datapoints are shown; colour‐day correspondence is in Supporting Information Table S2.

**Fig. 3:**
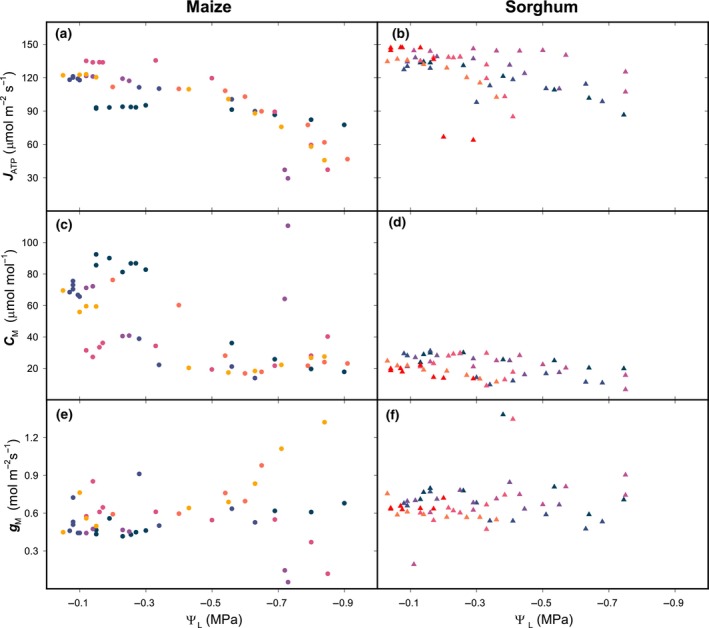
ATP production and mesophyll conductance. ATP production rate (*J*_ATP_, a, b) was calculated from Eqn 2 using assimilation, yield of PSII measured under ambient [O_2_] and low [O_2_], as well as light respiration estimated by curve fitting. CO_2_ concentration in the mesophyll (*C*_M_, c, d) was estimated through an oxygen and carbon isotopic discrimination model (Eqn 9) using measured values of Δ^13^C and Δ^18^O, *A*, and *C*_i_ (Fig. 2), which resulted in the mesophyll conductance to CO_2_ diffusion (gM) shown in e and f. Maize *n* = 6; sorghum *n* = 7 biological replicates; all datapoints are shown; colour‐day correspondence is in Supporting Information Table S2.

**Fig. 4:**
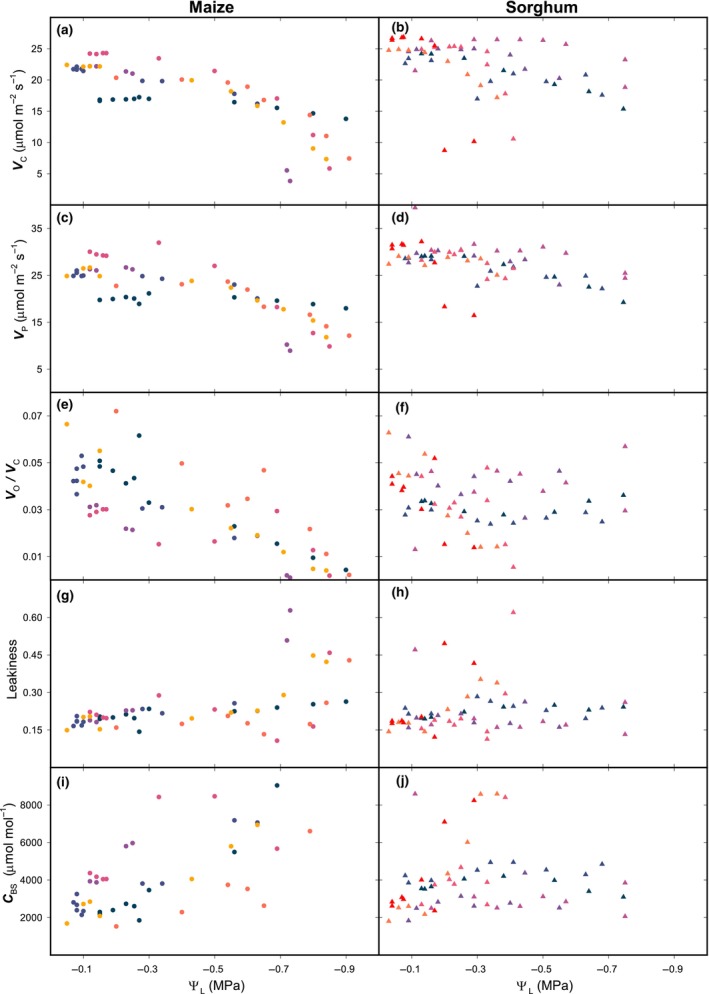
Rates estimated through a biochemical model of C_4_ assimilation. A biochemical model of assimilation (Eqns (Eqn 4), (Eqn 5), (Eqn 6), (Eqn 7), (Eqn 8)) was constrained with measured values of assimilation (Fig. 2a,b), and estimated values of *J*_ATP_ and *C*_M_ (Fig. 3). The dependence of bundle sheath conductance (*g*_BS_) upon Ψ_L_ was estimated by fitting modelled Δ^13^C (Eqn 9) to measured data (Fig. 2g,h). (a, b) Rubisco carboxylation rate, *V*_C_; (c, d) PEPC carboxylation rate, *V*_P_; (e, f) ratio between rates of Rubisco oxygenation *V*_O_ and *V*_C_; (g, h) leakiness (Φ, the ratio between the rate of CO_2_ retrodiffusing from the bundle sheath and PEP carboxylation rate *V*_P_); (i, j) CO_2_ concentration in the bundle sheath *C*_BS_. Maize *n* = 6; sorghum *n* = 7; all datapoints are shown; colour‐day correspondence is in Supporting Information Table S2.

In both maize and sorghum gas exchange quantities steadily declined. In detail, assimilation decreased (Fig. 2a,b), together with CO_2_ concentration in the substomatal cavity (*C*_i_, Fig. 2c,d), driven by stomatal closure (Fig. S2). Fluorescence‐derived yield of PSII (*Y*(II)) decreased (Fig. 2e,f), thereby lowering the derived total rate of ATP production under ambient O_2_ (*J*_ATP_, Fig. 3a,b), and the modelled rates of Rubisco and PEP carboxylation (*V*_C_ and *V*_P_, Fig. 4a–d). The trend of *C*_i_ was essentially mirrored by the CO_2_ concentration in the mesophyll (*C*_M_) (Fig. 3c,d), because mesophyll conductance to CO_2_ (*g*_M_) was unaffected by dehydration (Fig. 3e,f). Oxygen and carbon isotopic discrimination of CO_2_ showed opposing trends: while Δ^18^O decreased (Fig. 2i,j), Δ^13^C increased (Fig. 2g,h, see also as plotted against the ratio between internal and external CO_2_ concentration, *C*_i_ : *C*_a_, in Fig. S3). By contrast, the rate of CO_2_ diffusion out of the bundle sheath (Fig. 4g,h), and CO_2_ concentration in the bundle sheath (Fig. 4i,j) increased, thus suppressing photorespiration (Fig. 4e,f).

From these combined gas exchange fluorescence and isotopic discrimination data, bundle sheath conductances at full hydration (*g*_BS0_) were derived for each individual replicate (Table S2), and were, on average, similar for maize and sorghum (Table 3). Both in maize and sorghum *g*_BS_ significantly decreased with dehydration (*k* > 0). We tested whether the result was disproportionately influenced by responses observed under severe dehydration. Excluding the final one or two datapoints of each replicate had no significant effect (*P* = 0.8). To quantify the effect of model uncertainties, we performed 200 Monte Carlo iterations of fitting *g*_BS_, each using randomly generated combinations of model inputs from a normal distribution (Table S1). The resulting population of *g*_BS_ had a SD of *c*. 50% (Table S2), and that of *k* of *c*. 80%, thus caution is warranted when interpreting the value of *k*. However, upon testing the sign of *k*, we found a near‐zero probability of *k* being lower than zero, meaning that the pattern of decreasing *g*_BS_ with dehydration was not an artefact due to the specific parameterisation of the model.

To aid comparison between measured data and model output we computed the dimensionless elasticity to dehydration (η_Ψ_), which is the relative change in a variable per negative relative change in Ψ_L_ (Table 2). Although the trend was similar in both species, elasticity was generally greater in magnitude in maize, indicating more pronounced responses to dehydration. Among variables, *g*_BS_ had the most negative elasticity, that is decreasing the most with dehydration. In increasing order of elasticity, we found Rubisco relative oxygenation rate (*V*_O_/*V*_C_), CO_2_ concentration in the mesophyll, in the intercellular airspaces, and assimilation, sharing similar elasticity with Rubisco carboxylation rate. Elasticity was less negative than assimilation in variables related to electron transport, specifically, ATP production, PSII yield, and PEP carboxylation rates, along with Δ^18^O. Leakiness, Δ^13^C, and CO_2_ concentration in the bundle sheath had positive elasticity, that is they increased with dehydration, with *C*_BS_ increasing the most.

## Discussion

We set out to study the cause of the drop of C_4_ assimilation observed under dehydration. Concurrent monitoring of photosynthesis, fluorescence, and the isotopic composition of gases in real time presents technical challenges. These include the temperature sensitivity of the laser; the trade‐offs between flow rate and measurement sensitivity (Bellasio & Griffiths, 2014a); the low ratio between signal and noise due to the inherently low discrimination of C_4_ plants (Ubierna *et al*., 2018b); and uncertainty increasing with dehydration as stomatal closure decreases the ratio *C*_i_ : *C*_a_ (Farquhar, 1983). Surmounting these problems required years of experimental optimisation, assembling a bespoke thermostatic mount (Fig. S1), and the development of the dedicated laser management software, vinland. Data were input to a combined model of isotopic discrimination and C_4_ biochemistry, purposely formulated. Previous modelling approaches were unable to quantify the fraction of ATP used for the regeneration of PEP, which was frequently assumed constant. All else being equal, this fraction determines the ratio between the rates of PEP carboxylase and Rubisco, and the leak rate, calculated by their difference. This may explain the invariance of leakiness observed under drought by Sonawane & Cousins (2020). In our study, bundle sheath conductance was iteratively fitted using a novel strategy that allowed both leakiness and bundle sheath conductance to vary independently of the other (Table 1).

Our first hypothesis stated that assimilation would decrease due to hindered CO_2_ delivery to the mesophyll. Yet, we found no evidence supporting a response of mesophyll conductance to dehydration. This aligns with results for C_4_ sorghum exposed to drought for 12, 16 or 25 d obtained using an isotopic method (Sonawane & Cousins, 2020). By contrast, C_3_ plants exposed to weeks‐long drought often show a decrease in *g*_M_ (Flexas *et al*., 2004), but this is not a general rule. For instance, Hommel *et al*. (2014) found that one out of five C_3_ forest species maintained stable *g*_M_ under drought. These exceptions indicate that there may be factors other than dehydration itself causing reductions in *g*_M_. Our interpretation is that decreases in *g*_M_ observed under weeks‐long drought may not be due directly to dehydration, but, rather, they may be consequences of leaf ‘hardening’ in preparation for more severe stress. Tender leaves developed in hydrated conditions undergo coordinated acclimation strategies involving the dismantling of the photosynthetic machinery, thickening and lignifying walls, and the accumulation of defence metabolites. By contrast, leaves emerging during prolonged drought may employ alternative coping strategies, allowing for higher *g*_M_ compared to their well‐watered counterparts. This was shown, for instance, by Roig‐Oliver *et al*. (2020). Further support is offered by Pathare *et al*. (2020) who observed that, among 18 C_4_ grasses in a common garden, *g*_M_ was higher in accessions sourced from drier habitats, meaning that high *g*_M_ could be a beneficial adaptation to drought.

Our second hypothesis stated that assimilation would drop for a decrease in bundle sheath permeability to photosynthetic metabolites. Although bundle sheath permeability can be estimated indirectly *in vitro* (Weiner *et al*., 1988) and metabolite fluxes can be estimated through isotope labelling (Medeiros *et al*., 2022), those methods are both destructive. The method we used, the only one available for real‐time measurements during environmental manipulation, allows for the resolution of plasmodesmatal permeability to CO_2_ (*g*_BS_). We showed that *g*_BS_ decreased significantly with dehydration (*k* > 0, Table 3). Since CO_2_ and photosynthetic metabolites share the same diffusion path through plasmodesmata, our data support Hypothesis 2. The mechanisms leading to a decrease in *g*_BS_ remain unknown. The greater resistance may stem from a direct impact of dehydration on the physical process of diffusion, potentially involving the concentration‐driven increase in cytosol viscosity (Sidell & Hazel, 1987). Alternatively, it could be a consequence of turgor drop‐mediated contraction of cell walls, resulting in a reduction in cross section and an increase in plasmodesmatal length, or in moving intraplasmodesmatal components that form a barrier to diffusion closer together. Other possibilities include the deposition of callose around the neck (which occurs rapidly occurring after chilling (Bilska & Sowiński, 2010) and in response to pathogens (Petit *et al*., 2020)), or a combination of these factors.

Our innovative analysis of elasticity (Table 2) surpasses the conventional analysis of photosynthetic limitations by being quantitative and dimensionless. This allows for comparison between data and model output across species. We now interpret responses to dehydration, which are similar in maize and sorghum, in the context of accepting Hypothesis II. An initial decrease in metabolite conductance would hinder malate diffusion (Fig. 1b). The rate of NADPH delivery to the bundle sheath would consequently slow, creating a NADPH surplus in the mesophyll and a NADPH deficit in the bundle sheath. The latter would slow phosphoglycerate reduction and increase phosphoglycerate concentration in the bundle sheath, competitively inhibiting Rubisco carboxylation (Portis, 1992). Rubisco carboxylation's direct control over the rate of assimilation is supported by assimilation and Rubisco carboxylation rate having essentially the same elasticity (Table 2). This is consistent with the conclusion of Lal & Edwards (1996) that neither RuBP regeneration nor Rubisco capacity is limiting under water stress. The phosphoglycerate surplus in the bundle sheath would diffuse to the mesophyll and consume the NADPH surplus therein. The ATP surplus in the bundle sheath resulting from the reduced usage for phosphoglycerate reduction could be used for PEP regeneration either by pyruvate‐phosphate dikinase or by PEP carboxykinase (PEPCK) (Bellasio & Griffiths, 2014b), upholding PEP carboxylase activity. Indeed, the lower elasticity of the C_4_ cycle reactions (and of Photosystem II yield) than that of Rubisco carboxylation aligns with the findings of Lal & Edwards (1996) and Ghannoum *et al*. (2003). The mismatch between the slower malate diffusion and the invariant PEP carboxylase activity creates a surplus of oxaloacetate in the mesophyll. This would be diverted through transamination to aspartate (Bellasio, 2017), not requiring NADPH in the mesophyll, because the original NADPH surplus in the mesophyll is now consumed by the additional phosphoglycerate reduction. Aspartate would deliver CO_2_ to the mesophyll in parallel to malate, thus maintaining the effectiveness of CO_2_ delivery irrespective of the slower malate shuttle. This is supported by the experimental observation of the rate of Rubisco oxygenation having the second lowest elasticity and CO_2_ concentration in the bundle sheath having the highest elasticity of all quantities (Table 2). Once in the bundle sheath, aspartate would be converted back into oxaloacetate, and decarboxylated either through PEP carboxykinase or by malic enzyme after a futile reduction of oxaloacetate to malate (Furbank, 2011) which is supported by the observation that aspartate aminotransferase is also expressed in the absence of PEP carboxykinase activity (Schlüter *et al*., 2019). The former may be operating in maize, similarly to sugarcane, where PEP carboxykinase activity was upregulated under drought (Cacefo *et al*., 2019), while the latter may be more likely in sorghum.

While C_4_ assimilation appears susceptible in the timescale of hours‐to‐days, some C_4_ plants have shown the capacity to mitigate the impact of dehydration as drought progresses. For instance, in our previous studies with C_4_ *Eragrostis curvula*, the sensitivity of nonstomatal limitation to dehydration was high when dehydration was imposed over days (Quirk *et al*., 2019), and decreased remarkably and progressively over seasonal drought (Bellasio *et al*., 2022). The reasons for this delay in coping with drought in C_4_ plants is poorly understood; yet, the added complexity of C_4_ metabolism would require more time than for C_3_ to adjust. In natural environments, sudden drops in leaf water potential typically result from a combination of water depletion and an increase in leaf transpiration. Notably, both these factors ultimately hinge on the frequency of precipitation and the rate of dehydration, which are in turn determined by soil water retention capacity, temperature, and humidity, factors largely beyond the control of plants. Eventually, in some species, the benefits – whether direct, ancillary, or only indirectly related to C_4_ assimilation (Edwards *et al*., 2010) – may prevail, as demonstrated by a handful of successful C_4_ species gaining dominance over C_3_ grasses, making up the majority of the standing biomass in most of today's warm open ecosystems (Beerling & Osborne, 2006). However, it may be that all C_4_ plants have found strategies to adjust to gradual changes in water status. Any attempt to ameliorate drought tolerance in C_4_ plants or to convert a C_3_ crop into a C_4_ one will need a detailed understanding of those strategies.

## Competing interests

None declared.

## Author contributions

CB conceived the project, acquired funding and designed the experiment with contributions of JF. HS‐W provided isotopic methods. GDF provided laboratories and resources. CB performed the research with contributions of HS‐W. CB analysed the data, created, coded and ran the models. CB, HS‐W, JF and GDF wrote the paper.

## Supporting information


**Dataset S1** Raw data encompassing concurrent leaf water potential, CO_2_ and water concentration, and isotopic composition obtained under ambient and low‐O_2_ conditions in a fast dehydration experiment.

**Fig. S1** Experimental setup.
**Fig. S2** Stomatal conductance.
**Fig. S3** Isotopic response to *C*_i_ : *C*_a_.
**Notes S1** Solutions.
**Table S1** Monte Carlo analysis initial parameterisation and uncertainty distribution.
**Table S2** Error propagation analysis.
Please note: Wiley is not responsible for the content or functionality of any Supporting Information supplied by the authors. Any queries (other than missing material) should be directed to the *New Phytologist* Central Office.

## Data Availability

All data are available in Dataset S1.
