# Supplementary material for: Fast dehydration reduces bundle sheath conductance in C4 maize and sorghum
Source: New Phytol. 2024 Oct 25;244(6):2197–209. doi: 10.1111/nph.20167 (PMC11579431; doi:10.1111/nph.20167)
Supplement: Supplementary file 2 — Fig. S1 Experimental setup. Fig. S2 Stomatal conductance. Fig. S3 Isotopic response to C i : C a. Notes S1 Solutions. Table S1 Monte Carlo analysis initial parameterisation and uncertainty distribution. Table S2 Error propagation analysis. Please note: Wiley is not responsible for the content or functionality of any Supporting Information supplied by the authors. Any queries (other than missing material) should be directed to the New Phytologist Central Office. [file NPH-244-2197-s001.pdf]

Fast dehydration reduces bundle sheath conductance in C_4 maize and sorghum

Chandra Bellasio[1, 2, 3, 4], Hilary Stuart-Williams[4], Graham D Farquhar[4], and Jaume Flexas[3, 5]

Article acceptance date: 7 September 2024

Supporting Information

Note S1. Solutions

VC= (3*A)/4 + Jatp/20 + (17*Rlight)/20 - Rm/10 + (Cm*gBS)/10 - ((25*A^2)/4 - 5*A*Cm*gBS - 15*A*Gammas*Obs*gBS - (5*A*Jatp)/2 + (15*A*Rlight)/2 + 5*A*Rm + Cm^2*gBS^2 - 2*Cm*Gammas*Obs*gBS^2 + Cm*Jatp*gBS - 3*Cm*Rlight*gBS - 2*Cm*Rm*gBS + Gammas^2*Obs^2*gBS^2 - Gammas*Jatp*Obs*gBS - 17*Gammas*Obs*Rlight*gBS + 2*Gammas*Obs*Rm*gBS + Jatp^2/4 - (3*Jatp*Rlight)/2 - Jatp*Rm + (9*Rlight^2)/4 + 3*Rlight*Rm + Rm^2)^(1/2)/10 - (Gammas*Obs*gBS)/10

VP=Jatp/4 - A/4 - (3*Rlight)/4 + Rm/2 - (Cm*gBS)/2 + ((25*A^2)/4 - 5*A*Cm*gBS - 15*A*Gammas*Obs*gBS - (5*A*Jatp)/2 + (15*A*Rlight)/2 + 5*A*Rm + Cm^2*gBS^2 - 2*Cm*Gammas*Obs*gBS^2 + Cm*Jatp*gBS - 3*Cm*Rlight*gBS - 2*Cm*Rm*gBS + Gammas^2*Obs^2*gBS^2 - Gammas*Jatp*Obs*gBS - 17*Gammas*Obs*Rlight*gBS + 2*Gammas*Obs*Rm*gBS + Jatp^2/4 - (3*Jatp*Rlight)/2 - Jatp*Rm + (9*Rlight^2)/4 + 3*Rlight*Rm + Rm^2)^(1/2)/2 + (Gammas*Obs*gBS)/2

VO=Jatp/10 - A/2 - (3*Rlight)/10 - Rm/5 + (Cm*gBS)/5 - ((25*A^2)/4 - 5*A*Cm*gBS - 15*A*Gammas*Obs*gBS - (5*A*Jatp)/2 + (15*A*Rlight)/2 + 5*A*Rm + Cm^2*gBS^2 - 2*Cm*Gammas*Obs*gBS^2 + Cm*Jatp*gBS - 3*Cm*Rlight*gBS - 2*Cm*Rm*gBS + Gammas^2*Obs^2*gBS^2 - Gammas*Jatp*Obs*gBS - 17*Gammas*Obs*Rlight*gBS + 2*Gammas*Obs*Rm*gBS + Jatp^2/4 - (3*Jatp*Rlight)/2 - Jatp*Rm + (9*Rlight^2)/4 + 3*Rlight*Rm + Rm^2)^(1/2)/5 - (Gammas*Obs*gBS)/5

Table S1. Monte Carlo analysis initial parameterisation and uncertainty distribution
Uncertainty was deliberately set higher than the expected experimental error after Verbeeck *et al.* (2006) to account for potential interspecific differences or discrepancies between the original measurements and our experimental setup.

Symbol	Name	Units	Initial value	Uncertainty
γ^*	Half the reciprocal Rubisco specificity	μmol m^{-2} s^{-1}	0.000233	15% of the initial value
O_M	O2 concentration in the M	μmol mol^{-1}	210000	5% of the initial value
α	fraction of O_2 evolution in the bundle sheath	‰	0.15	15% of the initial value
J_{ATP} / GA	ATP cost of gross assimilation under low O_2	ATP / CO_2	5.4	0.1 ATP / CO_2
b_4'	combined fractionation of CO_2 ↔ HCO_3- conversion and PEPC fixation	‰	-5.7	10% of the |initial value|
δ_{GROWTH}	isotopic composition in the growth chamber	‰	-8.8	0.3 ‰
e	^{13}C fractionation during decarboxylation	‰	-6	10% of the |initial value|
b_3'	^{13}C fractionation during carboxylation by Rubisco	‰	30	10% of the initial value
s	^{13}C fractionation during leakage from BS to M	‰	1.8	10% of the initial value
f	^{13}C fractionation during photorespiration	‰	11.6	10% of the initial value
a_s	^{13}C fractionation during diffusion in air	‰	4.4	10% of the initial value
a_d	^{13}C fractionation during diffusion in aqueous solution	‰	0.7	10% of the initial value
h	^{13}C fractionation during hydration	‰	1.1	10% of the initial value

Table S2. Error propagation analysis

Two hundred Monte Carlo simulation runs were conducted with random parameterisation generated using the uncertainty distribution shown in Table S1, then k and $g_{BS\,0}$ were found iteratively by fitting Eqn 10 to measured data (Eqn 1, Figure 2G and 2H). Runs that successfully converged satisfied these imposed constraints: g_{BS}>0.00005; $g_{BS\,0}$<0.015; k>-0.02; k<0.08. The table provides the count of successful runs, the average value, and the dispersion represented by the standard deviation relative to the mean. Additionally, the probability of the mean being not greater than zero was calculated using a t-test. The colour refers to the symbols used in the main text.

		Date	Initial Value	Runs	Average	SD	$p(t)$	Colour
**Sorghum**	$g_{BS\,0}$	01/07/2019	0.00194	135	0.00232	47%	4E-52	
		02/07/2019	0.00179	142	0.00237	51%	2E-50	
		05/07/2019	0.00265	181	0.00285	41%	3E-78	
		07/07/2019	0.00225	170	0.00232	56%	1E-54	
		10/07/2019	0.00153	167	0.00234	53%	6E-57	
		11/07/2019	0.00212	163	0.00250	41%	7E-70	
		12/07/2019	0.00172	160	0.00288	68%	7E-42	
	k	01/07/2019	0.00194	136	0.00120	113%	4E-19	
		02/07/2019	0.000630	143	0.00139	91%	1E-26	
		05/07/2019	0.00207	181	0.00258	53%	4E-62	
		07/07/2019	0.000792	170	0.00065	170%	7E-13	
		10/07/2019	0.00152	166	0.00325	65%	6E-46	
		11/07/2019	0.00212	163	0.00367	50%	8E-59	
		12/07/2019	0.00167	159	0.00786	80%	2E-34	
**Maize**	$g_{BS\,0}$	16/07/2019	0.00219	200	0.00203	50%	3E-71	
		18/07/2019	0.00169	201	0.00612	73%	2E-48	
		23/07/2019	0.00172	167	0.00177	70%	2E-42	
		24/07/2019	0.00146	117	0.00129	84%	3E-24	
		25/07/2019	0.00321	152	0.00322	50%	7E-55	
		26/07/2019	0.00225	202	0.00394	32%	7E-106	
	k	16/07/2019	0.00244	200	0.00148	115%	2E-26	
		18/07/2019	0.000637	201	0.0286	88%	5E-38	
		23/07/2019	0.00234	167	0.00233	75%	3E-39	
		24/07/2019	0.00162	119	0.00102	128%	3E-14	
		25/07/2019	0.00350	153	0.00335	51%	2E-54	
		26/07/2019	0.00256	202	0.0312	55%	2E-66	

Figure S1. Experimental Setup for concurrent measurements of gas exchange, fluorometry, oxygen and carbon isotopic discrimination
1, gas mixing unit; 2, dew point control; 3, gas exchange analyser; 4, light; 5, plant; 6, water level regulator; 7, fluorometer (not visible); 8, Aerodyne Carbon Dioxide Isotope Monitor (in a thermostatic assembly); 9, Picarro Ring-Down Spectrometer; 10, Psychrometer; 11 water vapour calibration device.

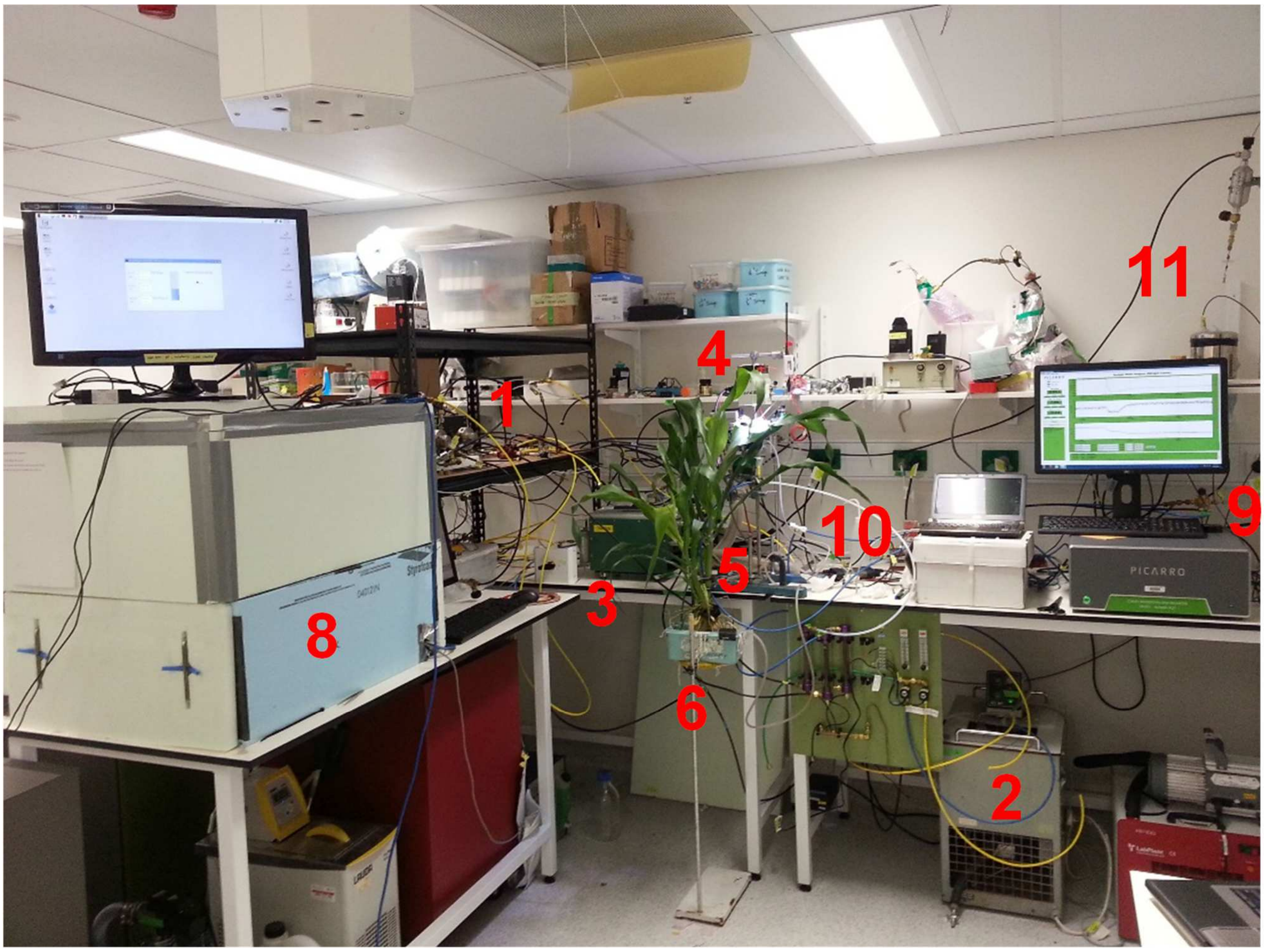

Figure S2. Stomatal conductance to CO_2 diffusion in C_4 maize and sorghum
Datapoints correspond to gas exchange data under ambient O_2 shown in Figure 2, the colour-date correspondence is detailed in Table S2.

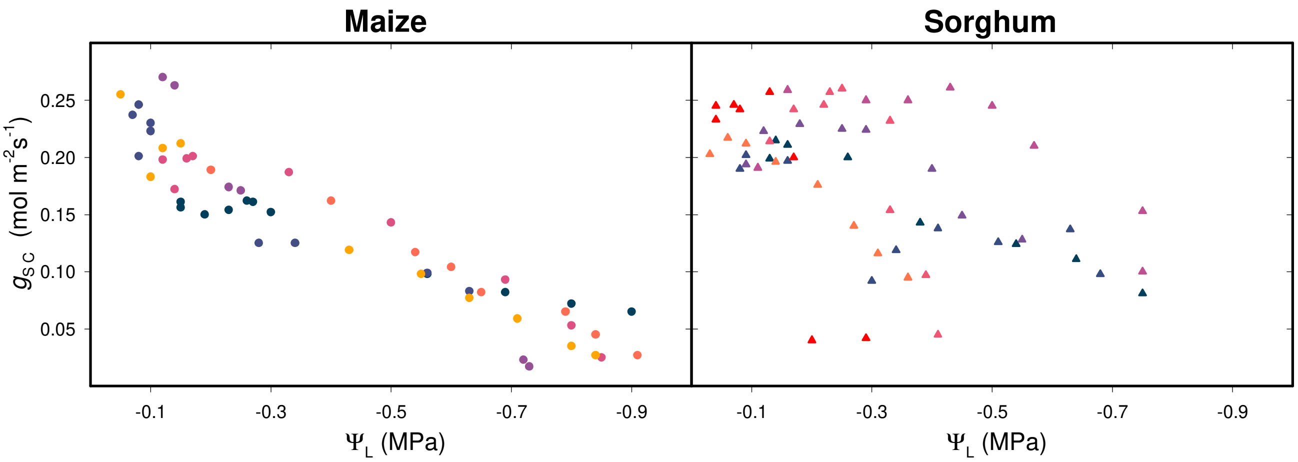

Figure S3. Stomatal and isotopic response to C_i/C_a

Relationship between stomatal conductance to CO_2 diffusion or carbon isotopic discrimination and the ratio between intercellular and external CO_2 concentration in C_4 maize and sorghum. Datapoints correspond to gas exchange data under ambient O_2 shown in Figure 2, the colour-date correspondence is detailed in Table S2.

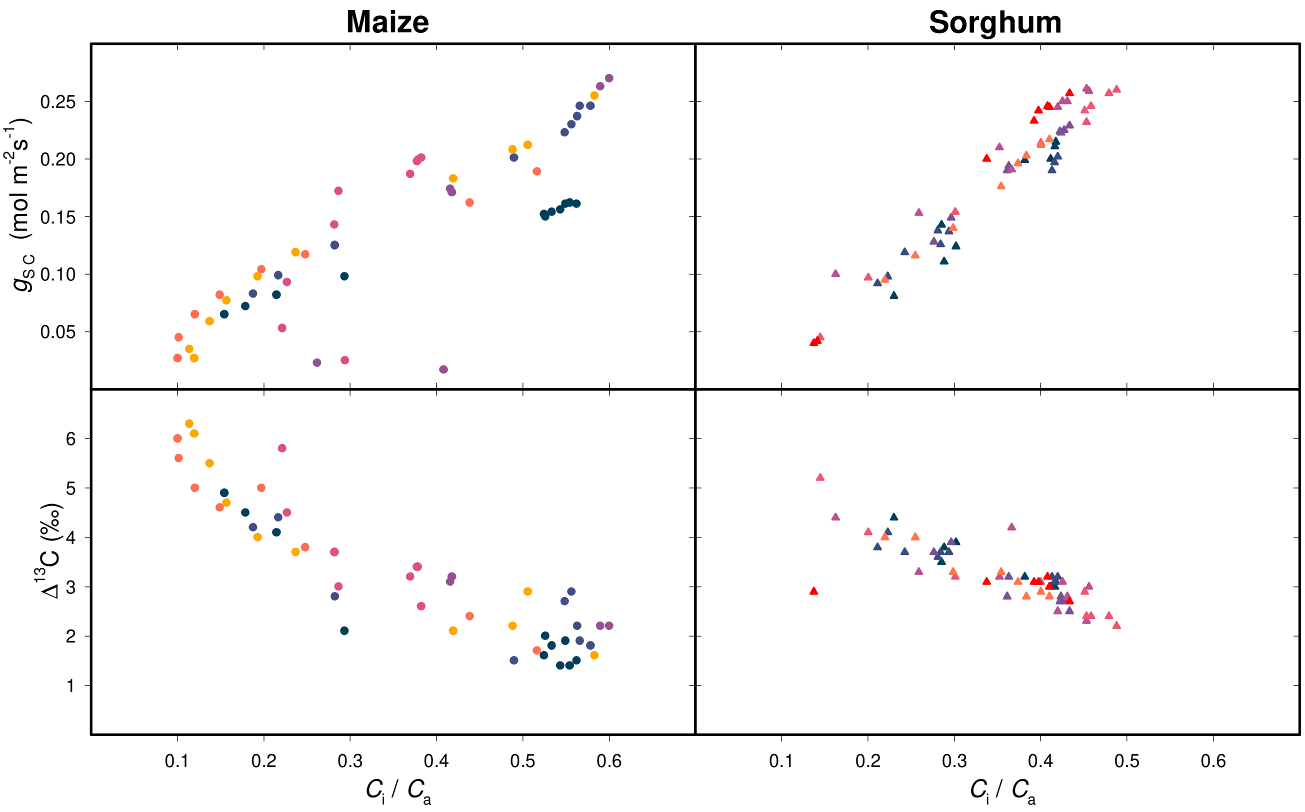

References

Verbeeck H, Samson R, Verdonck F, Lemeur R. 2006. Parameter sensitivity and uncertainty of the forest carbon flux model FORUG: a Monte Carlo analysis. *Tree Physiology* **26**(6): 807-817.
